# Reduction in hippocampal cholinergic neurostimulating peptide enhances memory impairment in *App^NL‐G‐F^* KI mice

**DOI:** 10.1002/alz.71531

**Published:** 2026-06-03

**Authors:** Yo Tsuda, Yuta Madokoro, Kengo Suzuki, Takuya Ooba, Toyohiro Sato, Yuto Uchida, Itsumi Nagai‐Arakawa, Takashi Saito, Hideaki Hara, Hideki Hida, Noriyuki Matsukawa

**Affiliations:** ^1^ Department of Neurology Nagoya City University Graduate School of Medical Sciences Nagoya Japan; ^2^ Department of Biofunctional Evaluation, Molecular Pharmacology Gifu Pharmaceutical University Gifu Japan; ^3^ Department of Neurocognitive Sciences Nagoya City University Graduate School of Medical Sciences Nagoya Japan; ^4^ Department of Neurophysiology and Brain Sciences Nagoya City University Graduate School of Medical Sciences Nagoya Japan

**Keywords:** Alzheimer's disease, *App^NL‐G‐F^* knock‐in, cholinergic dysfunction, hippocampal cholinergic neurostimulating peptide, hippocampal cholinergic neurostimulating peptide precursor protein

## Abstract

**INTRODUCTION:**

Whether cholinergic activity in the septohippocampal network affects cognitive dysfunction via hippocampal cholinergic neurostimulating peptide (HCNP) in Alzheimer's pathogenesis remains unclear.

**METHODS:**

An Alzheimer's pathogenesis by mutation in amyloid‐beta precursor protein gene (*App^NL‐G‐F^*) knock‐in (KI) and HCNP precursor protein conditional knockout (HCNP‐pp cKO) mouse model was generated, exhibiting both cholinergic dysfunction and amyloid pathogenesis. Theta power‐related cholinergic function and long‐term potentiation (LTP) were evaluated in *App^NL‐G‐F^* KI/HCNP‐pp cKO mice. Molecules associated with the cholinergic/glutamatergic neurons, amyloid beta (Aβ), and inflammation were examined.

**RESULTS:**

Reduced HCNP levels enhanced cognitive impairment, inhibiting theta power and LTP, although without accompanying pathological changes or inflammation. Decreased *N*‐methyl‐D‐aspartate receptor subunit 2A (NR2A), choline acetyltransferase (ChAT), and Vesicular acetylcholine transporter (VAChT) levels were observed in the hippocampus and ChAT in the medial septal nucleus (MSN) of *App^NL‐G‐F^* KI/HCNP‐pp cKO mice.

**DISCUSSION:**

Cholinergic dysfunction in HCNP‐pp cKO mice exacerbates cognitive dysfunction in *App^NL‐G‐F^* KI mice. The obtained mouse models are expected to be used to investigate cholinergic dysfunction and amyloid pathogenesis in AD.

## Highlights

- The effects of cholinergic activity on cognitive dysfunction were investigated.
- A mouse model with cholinergic dysfunction and amyloid pathogenesis was generated.
- Results showed that reduced hippocampal cholinergic peptide levels enhanced cognitive impairment.
- Cholinergic dysfunction exacerbated cognitive functioning in *App^*NL‐G‐F*^* KI mice.
- The model demonstrated cholinergic dysfunction and amyloid pathogenesis in Alzheimer's disease.

## 1 BACKGROUND

Lecanemab and donanemab are recently developed anti‐amyloid antibodies with disease‐modifying potential. However, their current clinical efficacy in symptomatic amelioration is approximately30%, considerably lower than the expected 60% reduction in amyloid beta (Aβ) plaque burden.^1^, ^2^ Epidemiological evidence has shown that some individuals can tolerate more Alzheimer's disease (AD)–related pathological changes than others, suggesting cognitive reserve,^3^ whereas multimodal intervention suggests the possibility of preserving neural activities in the form of clinical cognitive resilience.^4^, ^5^

To modulate network targeting for symptomatic therapy, AD researchers generally focus on septal cholinergic neurons under the cholinergic hypothesis^6^, ^7^ or hippocampal glutamatergic neurons under the glutamatergic hyperexcitability hypothesis.^8^ Neuronal modulators, such as donepezil, rivastigmine, galantamine, the acetylcholinesterase (AChE) inhibitor, and memantine, an antagonist of the *N*‐methyl‐d‐aspartate (NMDA) receptor, have been used extensively to ameliorate cognitive symptoms in patients with AD, with the aim to modulate and improve hippocampal glutamatergic activity. Aβ deposition has been found to induce cholinergic dysfunction in vivo in amyloid precursor protein (APP) model mice.^9^ Conversely, cholinergic dysfunction in the basal forebrain, nucleus basalis of the Meynert (NBM), and medial septal nucleus (MSN), has been reported to precede Aβ deposition in the AD brain.^7^ The mechanism by which cholinergic dysfunction precedes Aβ deposition and the precise relationship between dysfunction and deposition remain unknown. Clarifying this relationship may elucidate the symptomatic amelioration mechanism, including cognitive reserve and resilience, forming a solid foundation for drug discovery. However, no accurate model describing hippocampal glutamatergic impairment via cholinergic dysfunction in AD pathology has been developed.

Hippocampal cholinergic neurostimulating peptide (HCNP), originally purified from the soluble fraction of a rat hippocampus, has been reported to induce acetylcholine (ACh) synthesis in the MSN by increasing choline acetyltransferase (ChAT) production. This peptide sequence aligns with the *N*‐terminal region of the 21‐kDa precursor protein (HCNP‐pp), a 186‐amino acid protein reported to inhibit rapidly accelerating fibrosarcoma (Raf) and thus also known as Raf kinase inhibitory protein or phosphatidylethanolamine‐binding protein 1 (PEBP‐1).^10^, ^11^, ^12^ The expression of HCNP‐pp is significantly decreased in hippocampal pyramidal CA1 neurons during the earliest clinical phases of AD.^13^ To model AD‐related pathology, we demonstrated that ACh concentrations were decreased within the vesicular acetylcholine transporter (VAChT) in the hippocampus of HCNP‐pp conditional knockout (cKO) mice, resulting in reduced theta power and regional ChAT and inhibition of the slope of field excitatory postsynaptic potentials (fEPSPs) during long‐term potentiation (LTP).^14^, ^15^, ^16^ These data have suggested the adequacy of HCNP‐pp cKO mice as cholinergic functional impairment models describing septohippocampal interactions, even when no memory disturbance was observed in neurobehavioral assessment.^15^, ^17^ We previously reported that the cholinergic neuronal network from the MSN to the hippocampus can only functionally amplify hippocampal glutamatergic neural activity under insufficient glutamatergic neuronal network activity.^18^ Therefore, we hypothesized that insufficient hippocampal glutamatergic activity would confirm cholinergic dysfunction via HCNP reduction in an in vivo model. Additionally, Aβ oligomers in the hippocampus could functionally inhibit glutamatergic neuronal activity in the hippocampus.^18^ *App^NL‐G‐F^* mice are knock‐in (KI) mice that harbor the Swedish (KM670/671NL) and Beyreuther/Iberian (I716F) mutations with the Arctic (E693G) mutation in the *APP* gene leading to Aβ42 overproduction. Compared with previously reported AD‐model mice, *App^NL‐G‐F^* mice exhibit enhanced Aβ pathology, neuroinflammation, and memory impairment in an age‐dependent manner.^19^

To confirm the physiological effect of cholinergic dysfunction from the MSN to the hippocampus via HCNP reduction in the in vivo *APP* model and to generate an AD model of cholinergic dysfunction with amyloid pathology, we examined a cross of calcium/calmoduline‐dependent protein kinase II (CaMKII) Cre recombinase‐derived HCNP‐pp cKO and *App^NL‐G‐F^* KI mice in the current study. We performed behavioral testing and investigated the electrophysiological phenotype using theta power‐related cholinergic function and fEPSPs during LTP. We also assessed molecules associated with the cholinergic and glutamatergic terminals, amyloid pathogenesis, and inflammation via western blotting and immunohistochemistry.

## 2 MATERIALS AND METHODS

### 2.1 Animals

**RESEARCH IN CONTEXT**

1. **Systematic reviews**: We generated an Alzheimer's disease (AD) model of cholinergic dysfunction with amyloid pathology and examined the physiological effect of cholinergic dysfunction via hippocampal cholinergic neurostimulating peptide (HCNP), a cholinergic regulator. Reduced HCNP levels enhanced cognitive impairment, inhibiting theta power and long‐term potentiation (LTP) without pathological changes or inflammatory reactions in *APP^NL‐G‐F^* knock‐in mice.
2. **Interpretation**: Neuronal modulators clinically function symptomatic amelioration in patients with AD under cholinergic hypothesis. However, the relationship between cholinergic dysfunction and amyloid deposition remains unknown. No accurate model describing hippocampal glutamatergic impairment via cholinergic dysfunction in AD pathology has been developed. Our study established an Alzheimer's mouse model characterized by cholinergic dysfunction and amyloid pathology.
3. **Future directions**: To validate the importance of cholinergic function through HCNP on the mechanism by which cognitive resilience occurs, the pathological analysis of AD brains in correlation with cognitive function would be required.

The manuscript has been prepared in accordance with the ARRIVE 2.0 reporting guidelines. All animal experiments were approved by the Animal Care and Use Committee at Nagoya City University Graduate School of Medical Sciences (permit number 23‐034) and conformed to the guidelines for the use of laboratory animals published by the Japanese government (Law No. 105, October 1973). We used the appropriate number of animals for each experiment following the 3R (Replacement, Reduction, and Refinement) principles. The endpoint of this study was to delineate the precise mechanism of cognitive reserve or resilience, enabling the discovery of a new drug for cognitive amelioration.

**FIGURE 1:**
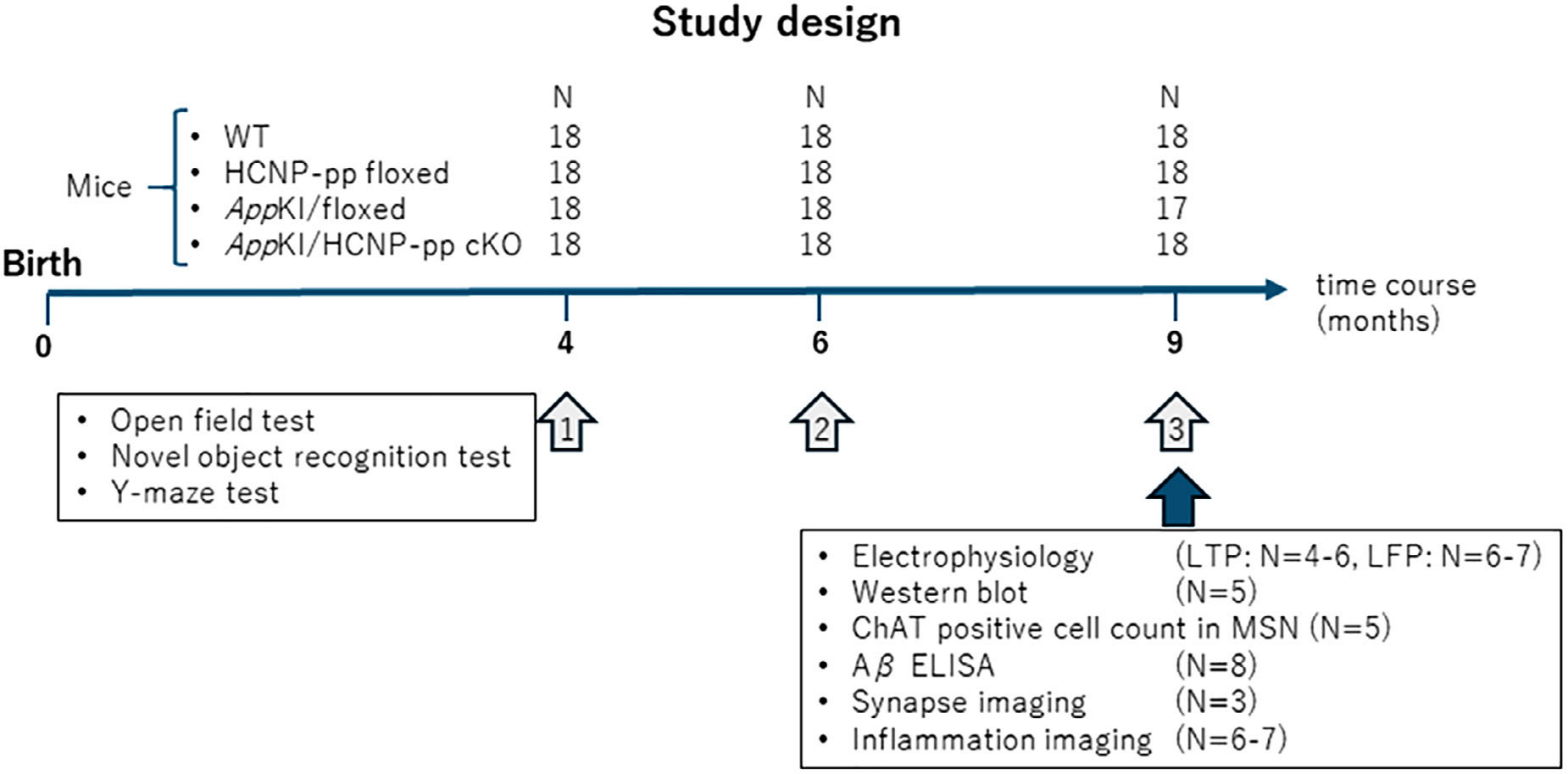
Schema of the experimental schedule. Behavioral tests were conducted in the same mice at 4, 6, and 9 months of age. Following behavioral assessments, each experiment, that is, electrophysiology, western blot, enzyme‐linked immunosorbent assay (ELISA), or immunohistochemistry, was performed independently. Here, *N* refers to the number of mice, and *n* refers to the number of slices or experimental measurements.

Homozygous HCNP‐pp‐floxed mice were generated as reported previously.^15^, ^17^ *App^NL‐G‐F^* mice are knock‐in mice that harbor Swedish (KM670/671NL) and Beyreuther/Iberian (I716F) mutations, along with the Arctic (E693G) mutation in the *APP* gene.^19^ *App^NL‐G‐F^* KI mice with HCNP‐pp cKO (*App^NL‐G‐F^* KI/HCNP‐pp cKO) were generated by mating heterozygous *App^NL‐G‐F^* KI mice carrying the homozygous HCNP‐pp‐floxed gene with heterozygous Cre recombinase transgenic mice driven by a CaMKII promoter also carrying the homozygous HCNP‐pp‐floxed gene, resulting in heterozygous *App^NL‐G‐F^* KI mice carrying homozygous HCNP‐pp‐floxed genes but no Cre recombinase transgene (*App^NL‐G‐F^* KI/floxed). The homozygous HCNP‐pp‐floxed gene (HCNP‐pp‐floxed) was utilized as a control, whereas C57BL/6J mice supplied by Japan SLC, Inc. (Hamamatsu, Japan), were used as wild‐type (WT) controls. All animals were housed under specific pathogen‐free conditions with a 12‐h light/dark cycle (lights on from 08:00 to 20:00) and were provided free access to food and water. A total of 71 male mice were used in the behavioral experiments (18 WT, 18 HCNP‐pp‐floxed, 17 *App^NL‐G‐F^* KI/floxed, and 18 *App^NL‐G‐F^* KI/HCNP‐pp cKO mice), whereas 46 female mice were utilized in the supplementary behavioral experiments (16 HCNP‐pp‐floxed, 14 *App^NL‐G‐F^* KI/floxed, and 16 *App^NL‐G‐F^* KI/HCNP‐pp cKO mice). After behavioral testing, 71 female mice at 11–12 months of age were included in the LTP experiments (18 WT, 18 HCNP‐pp‐floxed, 19 *App^NL‐G‐F^* KI/floxed, and 16 *App^NL‐G‐F^* KI/HCNP‐pp cKO mice), whereas 27 male mice at 11–12 months of age were included in the local field potential (LFP) experiments (7 WT, 7 HCNP‐pp‐floxed, 6 *App^NL‐G‐F^* KI/floxed, and 7 *App^NL‐G‐F^* KI/HCNP‐pp cKO mice). In addition, 28 male mice at 15–16 months of age were used in the supplementary LFP experiments (four groups of seven mice). Proteins from the hippocampi of 20 male mice at 9–10 months of age were assessed via western blot analysis and Aβ enzyme‐linked immunosorbent assay (ELISA; four groups of eight mice). Aβ accumulation was evaluated via immunohistochemical analysis of the brains of mice at 11–12 months of age (four groups of five mice). The brains of 20 male mice at 9–10 months of age were used for ChAT‐positive cell counting in the MSN (four groups of five mice), with some brain tissue samples used to evaluate inflammation (glial fibrillary acidic protein [GFAP]; four groups of seven mice, ionized calcium‐binding adapter molecule 1 [Iba1]N; four groups of six mice) via immunohistochemical analysis. Synapse imaging was also performed via immunohistochemical analysis of the brains of male mice at 9–10 months of age (four groups of three mice) (Figure 1).

### 2.2 Behavioral tests

To evaluate cognition and anxiety, the performance of model mice was investigated using open‐field locomotion, novel object recognition (NOR), and *Y*‐maze tests, conducted according to a previously described method.^15^ Behavioral testing was performed longitudinally in the same mice at 4, 6, and 9 months of age (Figure 1). In the NOR test, exploration time toward novel or familiar objects was quantified and the discrimination index (DI) was calculated as the ratio of (T_novel_ – T_familiar_) to (T_novel_ +T_familiar_). In the *Y*‐maze test, spontaneous alternation, defined as the proportion of sequential entries into all three arms, was used as an indicator of short‐term memory. All behavioral data were recorded by well‐trained researchers (T.O. and H.H.), who were blinded to the mouse genotypes.

### 2.3 Long‐term potentiation (LTP)

Glutamatergic neural functions in the hippocampus were also investigated, as described previously.^16^ Briefly, brains were rapidly removed under deep anesthesia, and 400‐µm‐thick transverse hippocampal slices were prepared using a vibrating slice cutter (Linear Slicer Pro 7, Dosaka, Kyoto, Japan) in an ice‐cold solution containing (mM): sucrose, 260; KCl, 3; NaH_2_PO_4_, 1.25; NaHCO_3_, 26; D‐glucose, 10, and MgCl_2_, 1 (pH 7.4), under continual bubbling with 95% O_2_ and 5% CO_2_. The hippocampal slices were then incubated for 30 min at 32°C in artificial cerebrospinal fluid (ACSF) containing (mM): NaCl, 125; KCl, 2.5; CaCl_2_, 2.4; MgCl_2_, 1; NaH_2_PO_4_, 1.25; NaHCO_3_, 25; and D‐glucose, 12.5. The ACSF was saturated with 95% O_2_ and 5% CO_2_ (pH 7.4). First, basal synaptic transmission in each slice was assessed by generating input–output (I/O) curves plotted against fiber volley (FV) amplitude for each genotype. The stimulus intensity that evoked 60% of the maximal response was then used for the subsequent experiments. Second, presynaptic function was evaluated using the paired‐pulse facilitation (PPF) index. The PPF index was calculated as the ratio of the second EPSP slope (R2) to the first EPSP slope (R1), with responses evoked at inter‐stimulus intervals of 25, 50, 75, 100, and 200 ms. For the electrophysiological LTP studies, the Schaffer collateral (SC) of CA3 was stimulated with repeated tetanus bursts (100 Hz, 1 s) to enhance the slope of fEPSPs in hippocampal LTP as preconditioning, and fEPSPs were recorded in the stratum radiatum (SR) of CA1. A stimulus–response curve was established at the beginning of each experiment with the stimulus intensity adjusted to evoke 30%–50% of the maximal response, corresponding to intensities of 20–40 µA. Under these experimental conditions, double‐tetanus stimulation (D‐TS) delivered with 2‐s intervals reliably induced robust fEPSP enhancement, whereas single‐tetanus stimulation (S‐TS) resulted in insufficient potentiation of the fEPSP slope. Furthermore, cholinergic activation via muscarinic M1 receptors was found to play a crucial role in the enhancement of fEPSP from S‐TS and D‐TS in both WT and HCNP‐pp cKO mice. Based on this, S‐TS or D‐TS was used as a preconditioning method on the SCs to evaluate the cholinergic function of glutamatergic activity‐inducing LTP, following a previously described technique.^16^, ^18^ LTP in the CA1 region was induced via S‐TS or D‐TS (0.1 ms pulse duration, 100 Hz for 1 s) of the SCs, whereas tetanus stimulation in the D‐TS was performed at 2‐s intervals. The average value of the fEPSP slope 50–60 min after tetanus stimulation was recorded and the ratio to the average value before induction was calculated as the LTP. Carbachol (CCh) or pirenzepine (Prz) was perfused along with ACSF for 10 min before LTP induction.

### 2.4 Local field potentials

As described previously, cholinergic neural function in the hippocampus was investigated in vivo as LFPs.^15^ Briefly, under anesthesia with 1.6 g/kg urethane, a 16‐channel silicon probe (NeuroNexus Technologies, Ann Arbor, MI, USA) was stereotactically inserted and advanced stepwise to the target position: anteroposterior (AP), −2 mm; L, −1.5 mm from the bregma; and dorsoventral (DV), 1.2 mm from the brain surface using a microcontroller. The neural activity of the pyramidal layer in the left CA1 was recorded at depths of 1100–1500 µm from the pia. Stereotactic coordinates were determined using the Paxinos and Franklin atlas.^19^ After application of a tail pinch immediately prior to electrical recording, all in vivo LFP recordings were digitally downsampled to 1 kHz using an Omniplex system (Plexon, Dallas, TX, USA) and filtered at a bandpass of 0.05–200 Hz. Proximity to the hippocampal pyramidal cell layer was determined by the: (1) depth of the probe, (2) presence of action potential discharge, and (3) phase reversal of the LFP at theta frequencies above and below the recording site. Each LFP, which was presumed to be in the pyramidal layer, was recorded for 1 min following tail pinch at four to seven locations per animal under urethane anesthesia. The recorded LFP data were then extracted using an offline sorter (Plexon) and the power spectral density of each LFP dataset was analyzed using NeuroExplorer (Plexon). The range of 3–12 Hz for the LFPs was extracted from the LFP as theta power.

### 2.5 Western blot analysis

Western blotting was performed following a previous method.^16^ In brief, hippocampal lysates containing 5–10 µg of protein were applied to Any kD Criterion TGX precast gels (Bio‐Rad, Hercules, CA, USA), and following sodium dodecyl sulfate (SDS)–polyacrylamide gel electrophoresis, the proteins were transferred onto polyvinylidene fluoride (PVDF) membranes (Amersham Hybond‐P PVDF 0.2; Cytiva, Cambridge, MA, USA). The membranes were then incubated overnight with 1:2000 rabbit polyclonal anti‐*N*‐methyl‐D‐aspartate receptor (NMDAR) subtype 1 (NR1) antibody (Sigma‐Aldrich, St. Louis, MO, USA), 1:5000 rabbit polyclonal anti‐NMDAR subtype 2A (NR2A) antibody (Merck‐Millipore, Burlington, MA, USA), 1:1000 rabbit polyclonal anti‐NMDAR subtype 2B (NR2B) antibody (Merck‐Millipore), 1:5000 rabbit polyclonal anti‐ α‐mino‐3‐hydrixy‐5‐methyl‐isoxazolepropionic Acid receptor (AMPAR) subtype 1 (GluA1) antibody (Merck‐Millipore), 1:1000 rabbit polyclonal anti‐AMPAR subtype 2/3 (GluA2/3) antibody (Merck‐Millipore), 1:2000 mouse monoclonal anti‐postsynaptic density 95 (PSD95) antibody (Merck‐Millipore), 1:500 goat polyclonal anti‐ChAT antibody (Merck‐Millipore), 1:5000 rabbit polyclonal anti‐ VAChT antibody (NOVUS Biological, Centennial, CO, USA), 1:1000 rabbit polyclonal anti‐ high‐affinity choline transporter 1 (CHT1) antibody (Merck‐Millipore), 1:200 000 rabbit polyclonal anti‐synaptophysin (Syn) antibody (Abcam, Cambridge, UK), 1:2000 rabbit polyclonal anti‐muscarinic receptor 1 (MR1) antibody (Sigma‐Aldrich), 1:5000 rabbit anti‐HCNP‐pp antibody,^15^ or 1:50 000 mouse monoclonal anti‐β‐actin antibody (Sigma‐Aldrich). The next day, membranes were labeled with the respective horseradish peroxidase‐conjugated anti‐rabbit, anti‐mouse, or anti‐goat immunoglobulin G (IgG) secondary antibodies. Bands were visualized using the ECL Prime Western Blotting Detection kit (Cytiva) or Select Western Blotting Detection kit (Cytiva) and imaged using ImageQuant LAS 4000 (Cytiva). Images were analyzed using the Amersham Imager 600 Analysis Software (Cytiva).

### 2.6 Immunohistochemical and histochemical studies

Immunohistochemistry was performed in accordance with a previous method.^14^, ^20^ After fixation with 4% PFA, 20‐µm‐thick sections were prepared from the frozen brains and incubated with 1:100 goat polyclonal anti‐ChAT antibody, 1:1000 mouse monoclonal anti‐GFAP antibody (Merck‐Millipore), 1:1000 rabbit polyclonal anti‐Iba1 antibody (Fujifilm, Osaka, Japan), 1:1000 mouse monoclonal anti‐synaptic vesicle glycoprotein 2A (SV2A) antibody (Santa Cruz Biotechnology, Dallas, TX, USA), 1:1000 rabbit polyclonal anti‐PSD95 antibody (Abcam), or 1:5000 rabbit anti‐HCNP‐pp antibody and detected with Alexa Fluor 488‐conjugated or Alexa Fluor 594‐conjugated donkey anti‐goat IgG, goat anti‐rabbit IgG, goat anti‐mouse IgG, or donkey anti‐rabbit IgG secondary antibodies. Images were then captured under a fluorescence (Axio Observer, Zeiss, Oberkochen, Germany) or A1Rsi laser confocal (Nikon, Tokyo, Japan) microscope.

For analysis of amyloid pathology, 5‐µm‐thick sections were prepared from paraffin‐embedded brains and fixed with 4% PFA before immunostaining with mouse monoclonal antibodies including 1:2000 anti‐N‐terminal Aβ (BAN50, Fujifilm, Tokyo, Japan), 1:100 anti‐C‐terminal Aβ40 (18580, Immuno‐Biological Laboratories, Gunma, Japan), and 1:100 anti‐C‐terminal Aβ42 (18582, Immuno‐Biological Laboratories). Citric acid treatment was utilized for antigen retrieval under BAN50 staining, and formic acid treatment (99% formic acid for 1 min at 25°C) was utilized for other processes. Histochemical analysis was performed using the method described in a previous study,^15^ in which paraffin‐embedded brain sections were stained with hematoxylin and eosin or using the Klüver–Barrera or Gallyas–Braak methods.

### 2.7 ChAT‐positive cell count in MSN

ChAT‐positive cells in the MSN were counted using a method described previously,^14^ with three consecutive sections 0.5 mm anterior from the bregma of each mouse used for counting the total number of positive cells. The area above the line connecting the lower ends of the anterior commissures on both sides was defined as the MSN. The number of ChAT‐positive cells in the MSN was counted using the ImageJ software (National Institutes of Health, Bethesda, MD, USA). Cells with ChAT intensity ≥11 and area ≥500 pixels were defined as ChAT‐positive, and the total number of ChAT‐positive cells was calculated using these data. After counting, ChAT‐positive cells were divided into two groups according to ChAT intensity: high concentration, >15; and low‐concentration, 11–15. The number of positive cells in each group was calculated from five mice, and the four groups were compared.

### 2.8 Enzyme‐linked immunosorbent assay (ELISA)

The soluble and insoluble Aβ fractions were separated following a previous method.^20^ In brief, Tris buffer containing 50 mM, Tris HCl (pH 7.6), 150 mM NaCl, and protease and phosphatase inhibitors was added to the hippocampal tissue, and the mixture was ground and centrifuged at 20 380 × *g* for 30 min at 4°C. The supernatant was then used as soluble Aβ. Pellets were redissolved in protein lysis buffer containing 30 mM, Tris HCl (pH 8.5), 7 M urea, 2 M thiourea, 3‐[(3‐cholamidopropyl)dimethylammonio]‐1‐propanesulfonate (CHAPS), and protease and phosphatase inhibitors, and centrifuged again at 20 380 × *g* for 30 min, with the resultant supernatant used as insoluble Aβ. The Aβ40 and Aβ42 in each fraction were then measured using an Aβ ELISA kit (Wako, Osaka, Japan) according to the manufacturer's instructions. When ELISA plates from different lots were used, aliquots from several identical bridging mouse samples were measured across all plates. Batch effects were corrected using these bridging samples, and the corrected values were used in the integrated analysis.

### 2.9 Measurement of synaptic numbers in hippocampus

For analysis, 20‐µm‐thick coronal sections were prepared, and one section was collected from each of three rostrocaudal positions at 100‐µm intervals (AP −2.4 mm, −2.3 mm, and −2.2 mm from bregma), resulting in three sections per mouse. Following double immunostaining with anti‐SV2A‐ and anti‐PSD95 antibodies, images were acquired using a confocal microscope (A1Rsi). A 40× objective lens was used, and *z*‐stack images were captured over an area of 318 × 318 µm with a *z*‐depth of 10 µm (0.425 µm per slice, 25 optical sections). Three‐dimensional reconstruction and analysis were performed using Imaris software (version 10.2.0, Bitplane, Zurich, Switzerland). For quantification, the number of SV2A‐ and PSD95‐positive puncta was first determined independently, and those located within 1 µm of each other were defined as co‐localized SV2A/PSD95 puncta.

### 2.10 Measurement of GFAP‐ and Iba1‐positive areas and volume

To evaluate inflammation in the hippocampus, immunostaining for GFAP and Iba1 and assessment was performed following a previous method.^20^ Images of the bilateral hippocampus were taken from three consecutive sections 2.5 mm posterior from bregma, with a total of six hippocampal images obtained from each mouse. To normalize the capture conditions, the laser power and exposure sensitivity were kept consistent while capturing the images. After visually extracting and trimming the whole hippocampus using the software, GFAP‐ and Iba1‐positive areas were measured using ImageJ. The average immune‐reactive–positive area from three mice in each group was calculated and compared among the four groups as a ratio to the background. For volumetric analysis, images were acquired under a confocal microscope (A1Rsi). The GFAP‐ or Iba1‐positive stained volume was quantified after three‐dimensional reconstruction using Imaris (Bitplane) and expressed as a ratio to the total tissue volume.

### 2.11 Data analysis

The primary unit of analysis was defined at the mouse level whenever possible (e.g., behavioral tests, western blotting, and ELISA), and all statistical analyses were performed accordingly. When multiple measurements were obtained from individual mice (e.g., tissue sections or electrophysiological recordings), linear mixed‐effects (LMEs) models were used to account for the hierarchical structure of the data and to avoid pseudoreplication; mouse ID was included as a random effect. Normality of the data distribution was assessed using the Shapiro–Wilk test, together with visual inspection of histograms, while homogeneity of variance was evaluated using Bartlett's test. Depending on distributional assumptions, group comparisons were performed using Student's *t*‐test or one‐way analysis of variance (ANOVA) followed by Tukey's post hoc test for normally distributed data with equal variances. When variance heterogeneity was detected, Welch's *t*‐test or Welch's ANOVA, with Holm‐adjusted pairwise comparisons, was applied. For non‐normally distributed data, the Kruskal–Wallis test, followed by Holm‐corrected Mann–Whitney *U* tests, was used. For electrophysiological analyses, basal synaptic transmission was evaluated using linear regression models with genotype, stimulus intensity (fiber volley amplitude), and their interaction as predictors. Presynaptic function assessed via PPF was analyzed using LME with genotype and inter‐stimulus interval as fixed effects and mouse ID as a random intercept. Longitudinal behavioral data from the NOR test were analyzed using LME, with group, month, and their interaction as fixed effects and mouse ID as a random intercept. In addition, an exploratory cross‐sectional analysis at 9 months of age was conducted using one‐way ANOVA. All statistical analyses were performed using the EZR software (Saitama Medical Center, Jichi Medical University, Shimono, Japan) and Python 3.10 (Python Software Foundation, Wilmington, DE, USA). Statistical significance was defined as *p* < 0.05, with Holm or false discovery rate (FDR) corrections applied for multiple comparisons where appropriate.

## 3 RESULTS

### 3.1 Reduced HCNP expression enhances memory dysfunction in *App^NL‐G‐F^* KI mice

**FIGURE 2:**
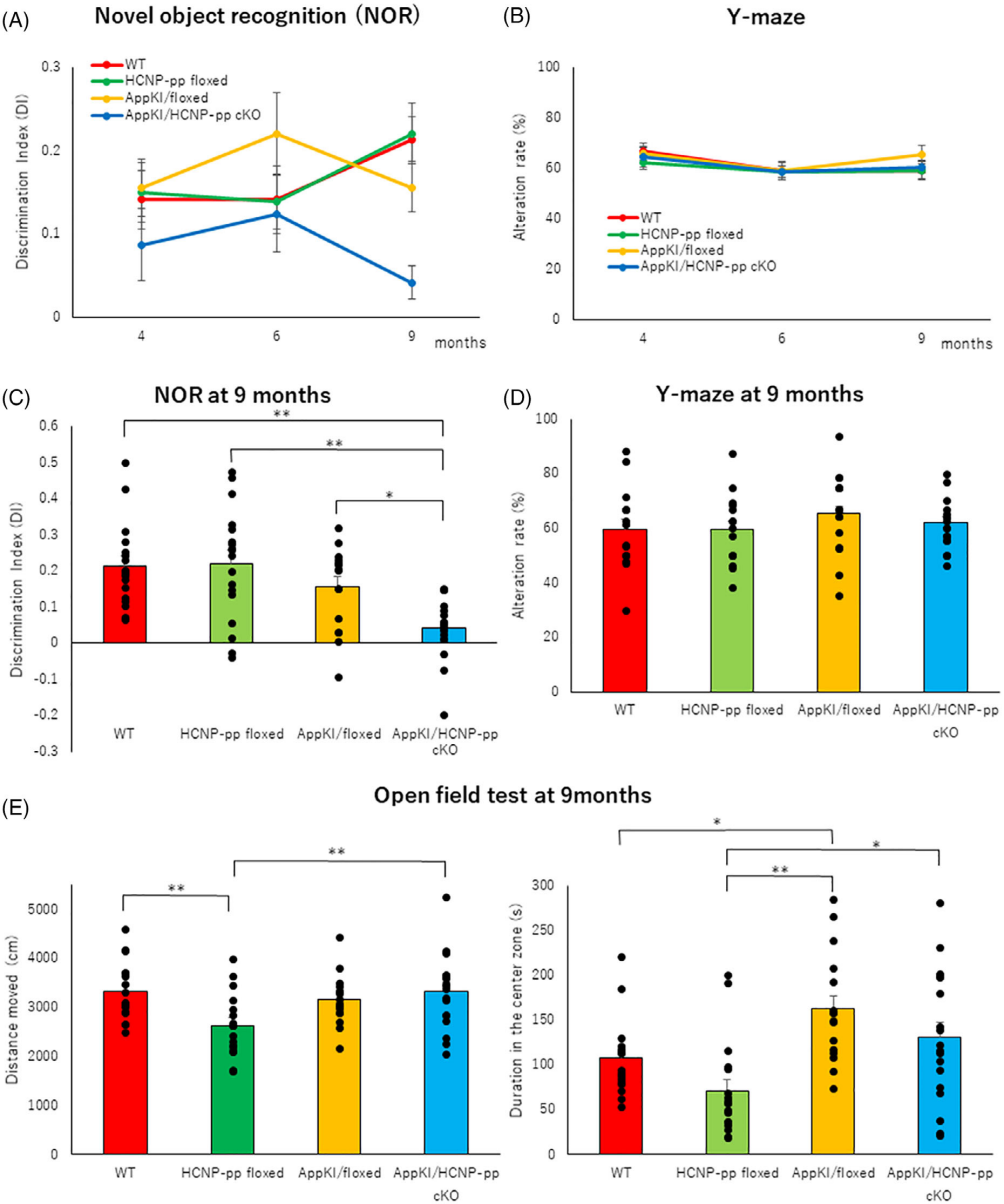
Behavioral evaluation. (A) Novel object recognition (NOR) test. On Day 3, after the two dimensional (2D) learning session, the right‐side object was replaced with a novel object. The exploration time toward the novel or familiar object was quantified, and the discrimination index (DI) was calculated as (*T*_novel_−*T*_familiar_) / (*T*_novel_ + *T*_familiar_). Linear mixed‐effects models revealed no significant differences among the four groups across the three time points. (B) In the *Y*‐maze behavioral test, linear mixed‐effects models similarly showed no significant differences among the four groups across the three time points. (C) At 9 months of age, the NOR test revealed that *App^NL‐G‐F^* KI/HCNP‐pp cKO mice exhibited a significant decrease in DI compared with wild type (WT), HCNP‐pp‐floxed, and *App^NL‐G‐F^* KI/floxed mice. (D) In contrast, the *Y*‐maze behavioral test at 9 months of age showed no significant differences among the four groups. (E) In the open field test at 9 months of age, the HCNP‐pp‐floxed group exhibited a significant decrease in total moving distance compared with that in the WT and *App^NL‐G‐F^* KI/HCNP‐pp cKO groups, indicating decreased activity levels. The duration spent in the center zone decreased in the HCNP‐pp‐floxed group. Male mice were used in all experiments (*N* = 18 WT, 18 HCNP‐pp floxed, 17 *App^NL‐G‐F^* KI/floxed, and 18 *App^NL‐G‐F^* KI/HCNP‐pp cKO). Longitudinal data were analyzed using a linear mixed‐effects model (A, B), whereas single‐time point data were analyzed using one‐way ANOVA. All data represent the mean ± standard error of the mean (S.E.M.). *p < 0.05, **p < 0.01 (raw data and statistical details are provided in Table S2).

To examine the effect of HCNP reduction on memory function and anxiety in *App^NL‐G‐F^* KI AD mouse models, we conducted behavioral experiments using *App^NL‐G‐F^* KI/HCNP‐pp cKO mice. Initially, using hematoxylin and eosin or Klüver–Barrera staining, we observed no fundamental morphological changes in the HCNP‐pp‐floxed, *App^NL‐G‐F^* KI/floxed, or *App^NL‐G‐F^* KI/HCNP‐pp cKO mice compared with WT mice. In addition, we detected no statistically significant differences in HCNP‐pp levels between WT and *App^NL‐G‐F^* KI mice at 9 months of age (Figure S1C, D, Table S1). However, subsequent NOR testing at 4, 6, and 9 months of age showed the first significant exacerbation of memory function in the *App^NL‐G‐F^* KI/HCNP‐pp cKO mice compared with that in 9‐month‐old WT, HCNP‐pp‐floxed, and *App^NL‐G‐F^* KI/floxed mice (Figure 2A, C, Table S2, Figure S2A, B, Table S3). We observed subsequent behavioral exacerbation in *App^NL‐G‐F^* KI/floxed mice at 12 months of age, resulting in levels similar to those in the *App^NL‐G‐F^* KI/HCNP‐pp cKO mice (Figure S2C, Table S3). However, the *Y*‐maze test revealed no significant exacerbation in *App^NL‐G‐F^* KI/HCNP‐pp cKO mice compared with that in 9‐month‐old WT, HCNP‐pp‐floxed, and *App^NL‐G‐F^* KI/floxed mice, indicating preserved overall function of the frontal cortex and hippocampus (Figure 2B, D, Table S2). These data suggested that reduced HCNP levels enhance the restricted memory dysfunction in *App^NL‐G‐F^* KI mice. In terms of locomotor activity, the total distance covered in the HCNP‐pp‐floxed mice differed from that of the WT and *App^NL‐G‐F^* KI/HCNP‐pp cKO mice at 9 months of age, with the duration in the center zone significantly reduced in the HCNP‐pp‐floxed mice, suggesting a decrease in exploratory behavior and increased anxiety compared with WT and *App^NL‐G‐F^* KI/HCNP‐pp cKO mice (Figure 2E, Table S2).

### 3.2 Reduced HCNP levels inhibit hippocampal glutamatergic neuronal activation via decreased theta power in *App^NL‐G‐F^* KI mice

**FIGURE 3:**
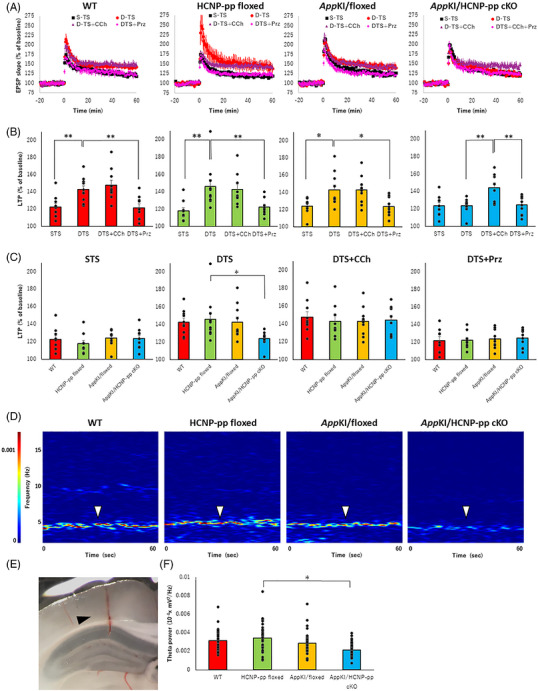
Electrophysiological assessment. (A) In the WT, HCNP‐pp‐floxed, *App^NL‐G‐F^* KI/floxed, and *APP^NL‐G‐F^* KI/ HCNP‐pp cKO groups, time course of field excitatory postsynaptic potentials (fEPSPs) were shown. (B) In WT, HCNP‐pp floxed, and *APP^NL‐G‐F^* /floxed groups, the fEPSPs were enhanced during long‐term potentiation (LTP) by double tetanus stimulation (D‐TS) compared with those by single tetanus stimulation (S‐TS). This effect disappeared following treatment with pirenzepine (Prz), an muscarinic receptor 1 (MR1) blocker, suggesting that it was under the influence of cholinergic neurons. However, no significant increase in fEPSP levels was observed with D‐TS alone compared with S‐TS in the *App^NL‐G‐F^* KI/HCNP‐pp cKO group during LTP. Treatment with carbachol (CCh), a cholinergic agonist, enhanced the fEPSPs induced by D‐TS in the *App^NL‐G‐F^* KI/HCNP‐pp cKO group to a level similar to that induced by D‐TS in the other three groups. This effect disappeared after Prz treatment. (C) When comparing the four groups, fEPSPs induced by D‐TS were significantly lower in the *App^NL‐G‐F^* KI/HCNP‐pp cKO group than in the HCNP‐pp‐floxed group. In contrast, fEPSPs induced by S‐TS, D‐TS in the presence of CCh or D‐TS with Prz did not differ significantly among the four groups. These data suggested that cholinergic nerve activity was reduced in the *App^NL‐G‐F^* KI/HCNP‐pp cKO group compared with that in the other three groups. Female mice aged 11–12 months were included in this study. *N* (mouse) = 4–6, *n* (slice) = 8–10. All data are presented as the mean ± S.E.M. *p < 0.05, **p < 0.01 (raw data and statistical details are provided in Table S5). For analysis of local field potential (LFP), an electrode was inserted into the CA1 pyramidal layer of the hippocampus under urethane anesthesia (E, arrowhead) and LFP was measured for 1 min following tail pinch. Representative spectrograms for each group are shown (D). Theta waves from 3 to 12 Hz were observed predominantly in all groups (white arrowheads). The in vivo study also confirmed a decrease in cholinergic nerve activity in the *App^NL‐G‐F^* KI/ HCNP‐pp cKO group compared with that in the HCNP‐pp floxed group (F). Male mice at 9 months of age were included in this study. *N* (mouse) = 7, *n* (insertion site) = 35–37. All data are presented as the mean ± S.E.M. Data were analyzed using a linear mixed‐effects model with genotype as a fixed effect and mouse identification document (ID) as a random intercept to account for multiple slices or insertion sites per mouse. *p < 0.05, **p < 0.01 (raw data and statistical details are provided in Table S5).

First, we confirmed that basal synaptic transmission and presynaptic function did not differ significantly among the four groups, as assessed by input–output curves and paired‐pulse facilitation (PPF), after adjustment for multiple comparisons using both Holm and Benjamini–Hochberg FDR procedures (Figure S3A, B; Table S4). Next, to examine the extent to which HCNP reduction accelerates memory dysfunction, we electrophysiologically investigated the fEPSP slope during LTP, which is related to memory function through hippocampal glutamatergic neuronal functioning. We have previously reported the experimental conditions required for evaluation of the cholinergic network function in the hippocampus during LTP. The enhancement of the fEPSP slope from S‐TS to D‐TS during LTP as preconditioning may be induced by cholinergic neuronal activity via muscarinic receptor 1 (MR1).^16^, ^18^, ^21^ In the present study, the fEPSP slope during LTP was enhanced from S‐TS to D‐TS in WT, HCNP‐pp‐floxed, and *App^NL‐G‐F^* KI/floxed mice, whereas the slope was never enhanced by CCh, a cholinergic agonist, consistent with the results of previous studies.^18^, ^21^ However, the enhancement was impaired by Prz, an antagonist against MR1 (Figure 3A, B, Table S5). Although we observed no enhancement of the fEPSP slope from S‐TS to D‐TS during LTP in *App^NL‐G‐F^* KI/HCNP‐pp cKO mice, the fEPSP slope by D‐TS was significantly enhanced by CCh administration (Figure 3A, B, Table S5), which was conversely inhibited by Prz. Among the four groups, the fEPSP slopes induced by D‐TS were significantly lower in *App^NL‐G‐F^* KI/HCNP‐pp cKO mice than in HCNP‐pp‐floxed mice. In contrast, fEPSP slopes induced by S‐TS, D‐TS in the presence of CCh, or D‐TS with Prz did not differ significantly among the four groups (Figure 3C, Table S5). These data suggested that cholinergic neuronal dysfunction may be involved in the low hippocampal glutamatergic activity that is induced by D‐TS in *App^NL‐G‐F^* KI/HCNP‐pp cKO mice.

To directly confirm cholinergic dysfunction in the hippocampus of *App^NL‐G‐F^* KI/HCNP‐pp cKO mice, we measured the LFP for 1 min following insertion of the electrode into the CA1 pyramidal layer of the hippocampus and tail pinch under urethane anesthesia (Figure 3D, E). The theta power of 3–12 Hz was significantly decreased in *App^NL‐G‐F^* KI/HCNP‐pp cKO mice compared with that in HCNP‐pp‐floxed mice (Figure 3D, F, Table S5). Combined with LTP and LFP results, these data suggested cholinergic dysfunction from the MSN to the hippocampus in *App^NL‐G‐F^* KI/HCNP‐pp cKO mice. At 15–16 months of age, however, we observed no significant difference in LFP among the four groups (Figure S3, Table S4).

### 3.3 Involvement of presynaptic cholinergic dysfunction via ChAT and VAChT in hippocampal glutamatergic reduction in *App^NL‐G‐F^* KI/HCNP‐pp cKO mice

**FIGURE 4:**
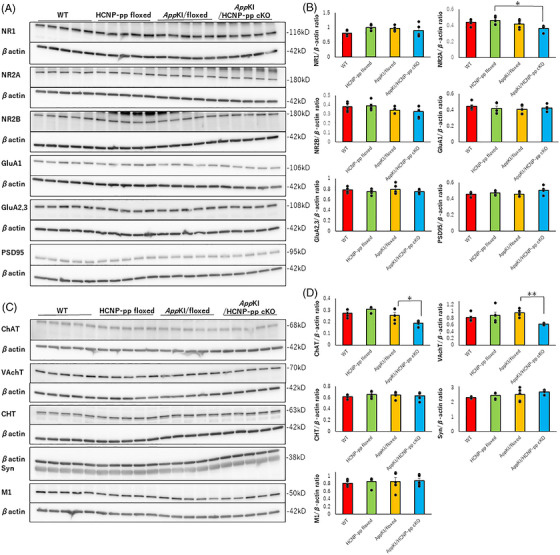
Biochemical evaluation of relevant molecules. (A) Proteins related to glutamatergic neurons and postsynapse were analyzed using western blotting. (B) After normalization with the β‐actin level, only the level of *N*‐methyl‐D‐aspartate receptor (NMDAR) subtype 2A (NR2A) was decreased in the *App^NL‐G‐F^* KI/ HCNP‐pp cKO group compared with that in the HCNP‐pp‐floxed group; however, no statistically significant difference was observed in the wild type (WT) and *App^NL‐G‐F^* KI/floxed groups. (C) Proteins related to cholinergic neurons and presynapse were analyzed using western blotting. (D) The ratios of choline acetyltransferase (ChAT) and vesicular acetylcholine transporter (VAChT) to β‐actin were decreased in the *App^NL‐G‐F^* KI/HCNP‐pp cKO group compared with those in the *App^NL‐G‐F^* KI/floxed group. No statistically significant differences were observed in the levels of choline transporter 1 (CHT1), synaptophysin (Syn), and muscarinic receptor 1 (MR1), with effect sizes being small. Male mice aged 9–10 months were included in this study. *N* (mouse) = 5. Statistical analysis was performed using one‐way ANOVA or Welch's ANOVA. All data are presented as the mean ± S.E.M. *p < 0.05, **p < 0.01 (raw data and statistical details are provided in Figure S4, Table S6).

To confirm the mechanism underlying glutamatergic dysfunction in the hippocampus of *App^NL‐G‐F^* KI/HCNP‐pp cKO mice, we quantified molecules associated with postsynaptic glutamatergic neurons using five independent mice per group. The level of NR2A, a subunit of the *N*‐methyl‐d‐aspartate (NMDA) receptor, was significantly decreased in the hippocampus of *App^NL‐G‐F^* KI/HCNP‐pp cKO mice compared with that in HCNP‐pp‐floxed mice; however, we observed no significant difference in the levels of NR1, NR2B, GluA1, GluA2/3, and PSD95 among the four groups, with effect sizes being small (Figure 4A, B; Table S6; Figure S4A–L). Similarly, we also examined the molecules associated with presynaptic cholinergic neurons, that is, ChAT, VAChT, CHT1, Syn, and postsynaptic M1R, and found significant reductions in ChAT and VAChT levels in the hippocampus of *App^NL‐G‐F^* KI/HCNP‐pp cKO mice compared with those in *App^NL‐G‐F^* KI/floxed mice (Figure 4C, D, Table S6, Figure S4M–V). In these experiments, we observed no statistically significant change in M1R levels among the four groups; however, a previous study revealed a significant increase in M1R levels in the hippocampus of HCNP‐pp cKO mice compared with that in HCNP‐pp floxed mice at 10–12 months of age.^16^ Based on electrophysiological data, these western blot data suggested that presynaptic cholinergic dysfunction, which partly involves postsynaptic glutamatergic receptors, is mainly involved in the glutamatergic electrophysiological impairment in the hippocampus of *App^NL‐G‐F^* KI/HCNP‐pp cKO mice.

### 3.4 Decrease in ChAT‐positive neurons with high concentrations of MSN in *App^NL‐G‐F^* KI/ HCNP‐pp cKO mice

**FIGURE 5:**
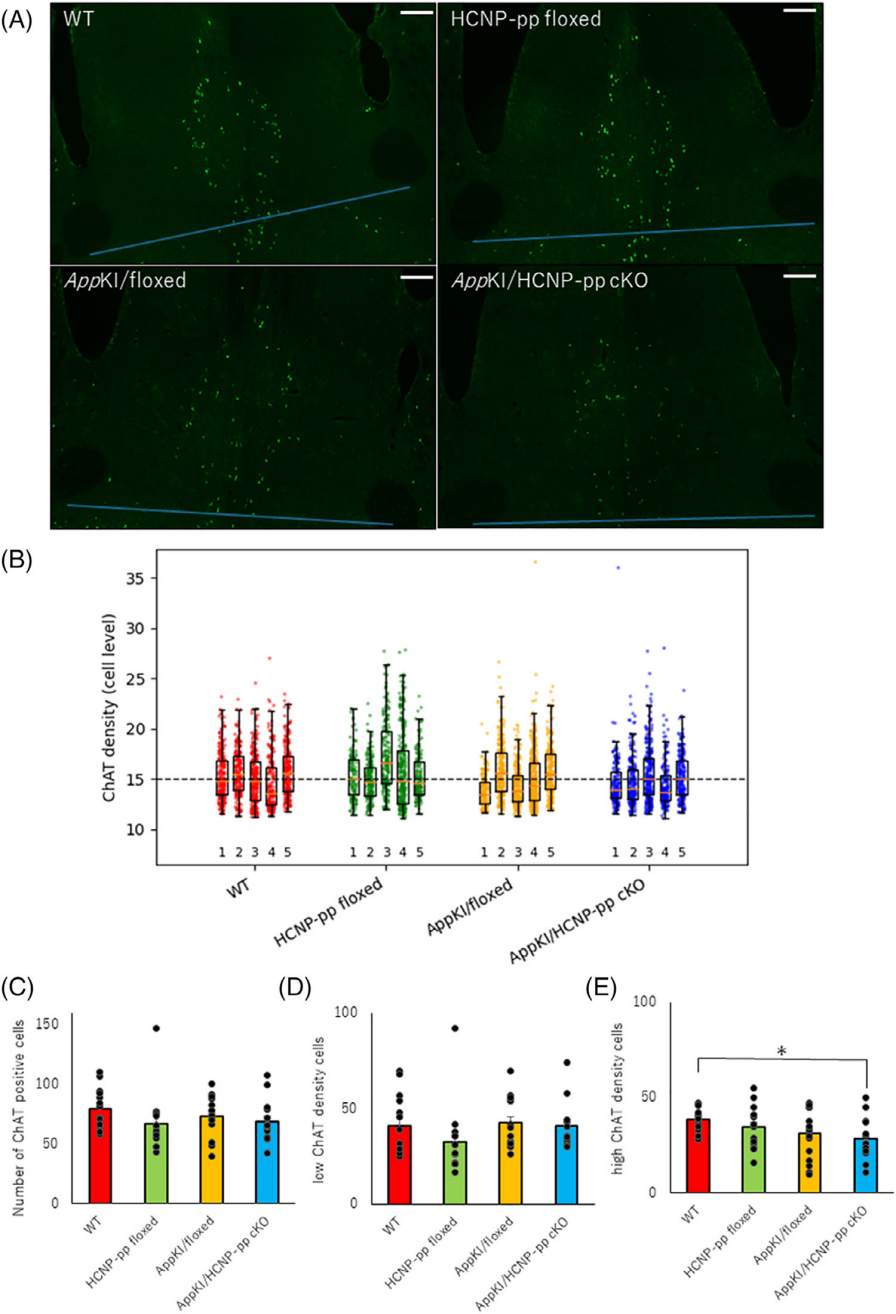
Count of choline acetyltransferase (ChAT)‐positive cells and evaluation of ChAT densities in medial septal nucleus (MSN). (A) To assess the number of ChAT‐positive cells in the MSN, frozen sections were immunostained with an anti‐ChAT antibody and positive cells above the blue line connecting the lower ends of the anterior commissures on both sides were counted. Scale bar = 200 µm. (B) ChAT‐positive cells were divided into two groups according to intensity: high‐concentration, >15; low‐concentration, 11–15. Distributions of ChAT concentrations in all ChAT‐positive cells in the four groups are shown. (C–E) Bar graphs revealing the count of the three degrees of ChAT concentration in the four groups. A significant reduction was observed in the concentration of ChAT‐positive cells in the *App^NL‐G‐F^* KI/HCNP‐pp cKO group compared with that in the wild type (WT) group, whereas no significant change in the total number of ChAT‐positive cells was observed. These data suggested lower amounts of ChAT in the MSN of *App^NL‐G‐F^* KI/HCNP‐pp cKO mice than those in WT mice. Male mice aged 9–10 months were used in both cases. *N* (mouse) = 5, *n* (slice) = 14. All data are presented as the mean ± S.E.M. Data were analyzed using a linear mixed‐effects model with genotype as a fixed effect and mouse identification document (ID) as a random intercept to account for multiple slices per mouse. *p < 0.05, **p < 0.01 (raw data and statistical details are provided in Table S7).

We previously reported that HCNP administration into the ventricles enhanced the ChAT‐positive axonal length and number of branches in the stratum oriens of CA1 in HCNP‐pp cKO and WT mice.^22^ Hence, we counted the number of ChAT‐positive neurons in the MSN to investigate whether HCNP has a trophic effect on cholinergic neurons in the MSN. We observed no significant differences among the four groups, suggesting no trophic effect of HCNP on cholinergic neurons in the MSN under these experimental conditions (Figure 5A, C, Table S7). Next, to confirm a reduction in the ChAT level in the MSN, we formed two groups based on the density using ImageJ, with ≥11–<15 considered low concentration and ≥15 considered high concentration. The number of cells with high ChAT concentrations in the MSN was significantly reduced in *App^NL‐G‐F^* KI/HCNP‐pp cKO mice compared with that in WT mice, whereas we observed no significant difference in the number of ChAT‐positive cells among the four groups (Figure 5B‐E, Table S7). These data suggested that HCNP enhances ChAT synthesis, consistent with our previous study.^11^

### 3.5 No significant exacerbation of Aβ‐related pathology in *App^NL‐G‐F^* KI mice due to reduced HCNP expression

**FIGURE 6:**
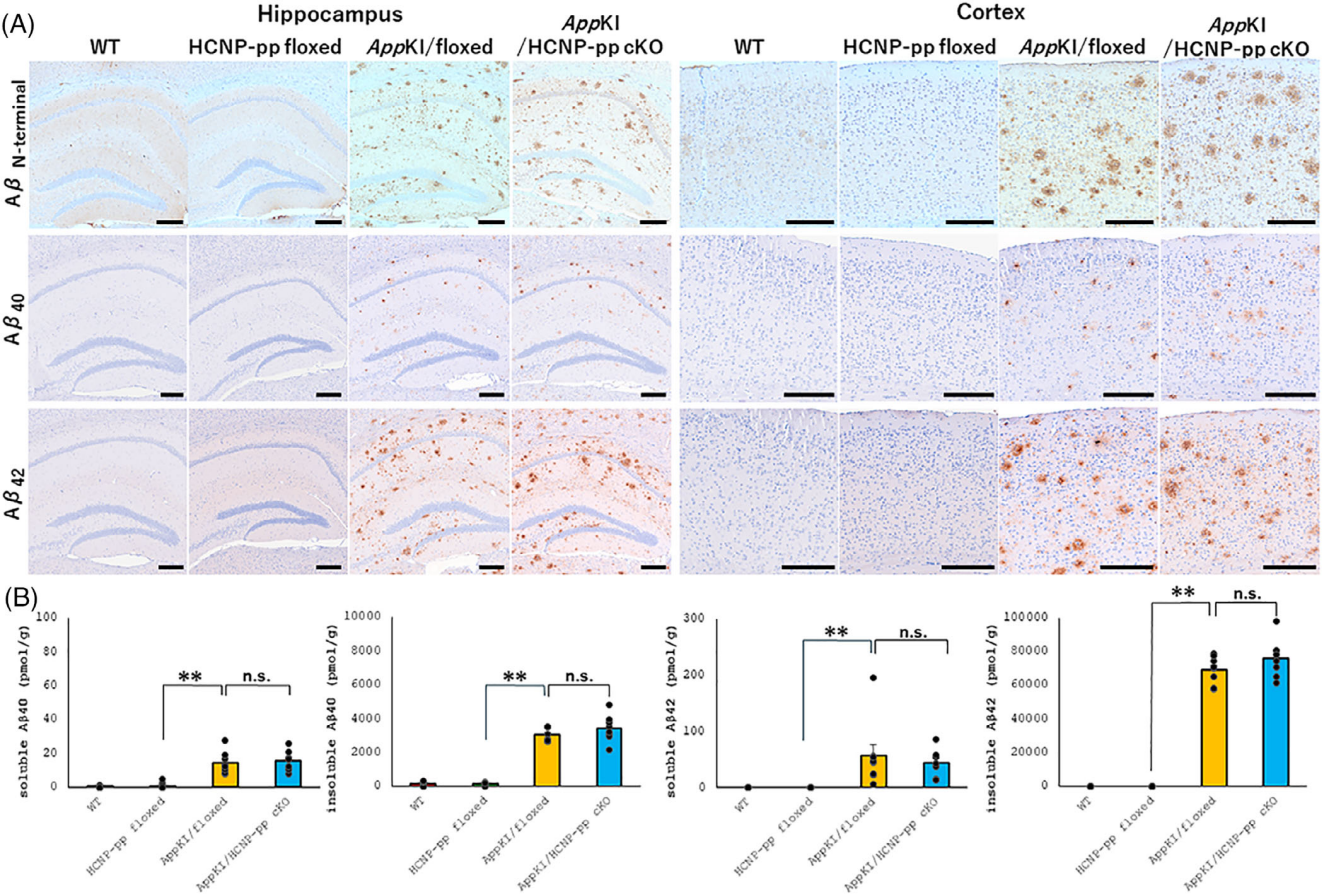
Evaluation of amyloid beta (Aβ) change. (A) Immunostaining of paraffin specimens of mice at 9–10 months of age revealed numerous Aβ plaques in the *App^NL‐G‐F^* KI/floxed and *App^NL‐G‐F^* KI/HCNP‐pp cKO groups. Scale bar = 200 µm. (B) The levels of Aβ40 and Aβ42 were quantified in both soluble and insoluble fractions of the hippocampus using sandwich enzyme‐linked immunosorbent assay (ELISA). Although Aβ accumulation was observed in the *App^NL‐G‐F^* KI/floxed and *App^NL‐G‐F^* KI/HCNP‐pp cKO groups at 9–10 months of age, no significant difference was observed between the groups. *N* (mouse) = 8. Statistical analysis was performed using the Kruskal–Wallis test. All data are presented as the mean ± S.E.M. **p < 0.01 (raw data and statistical details are provided in Table S8).

Cholinergic dysfunction in the MSN induced by Aβ accumulation and inflammation fundamentally precedes early pathological events in the development of cholinergic neural degeneration.^23^, ^24^, ^25^ Of interest, cholinergic cell death caused by saporin in the MSN could potentially impair Aβ clearance by intramural periarterial drainage as a result of decreased vascular reaction.^26^ Next, to examine whether the cholinergic dysfunction in the MSN that is attributable to HCNP reduction may exacerbate Aβ pathology, we performed immunohistochemical and biochemical analyses. Our immunohistochemical results showed no significant exacerbation in amyloid plaque accumulation in the hippocampus and frontal cortex of *App^NL‐G‐F^* KI/HCNP‐pp cKO mice compared with that in *App^NL‐G‐F^* KI/floxed mice as well as no Aβ pathology in the hippocampus and frontal cortex of WT and HCNP‐pp‐floxed mice (Figure 6A). The biochemical analysis showed no significant difference between the *App^NL‐G‐F^* KI/HCNP‐pp cKO mice and *App^NL‐G‐F^* KI/floxed mice in terms of both Aβ40 and Aβ42 (Figure 6B, Table S8). The obtained data and electrophysiological results suggested that HCNP reduction may exacerbate the results in behavioral experiments without significant changes in Aβ accumulation in the hippocampus of *App^NL‐G‐F^* KI/floxed mice.

### 3.6 No significant decrease in synaptic number in the CA1 region in *App^NL‐G‐F^* KI mice due to reduced HCNP expression

**FIGURE 7:**
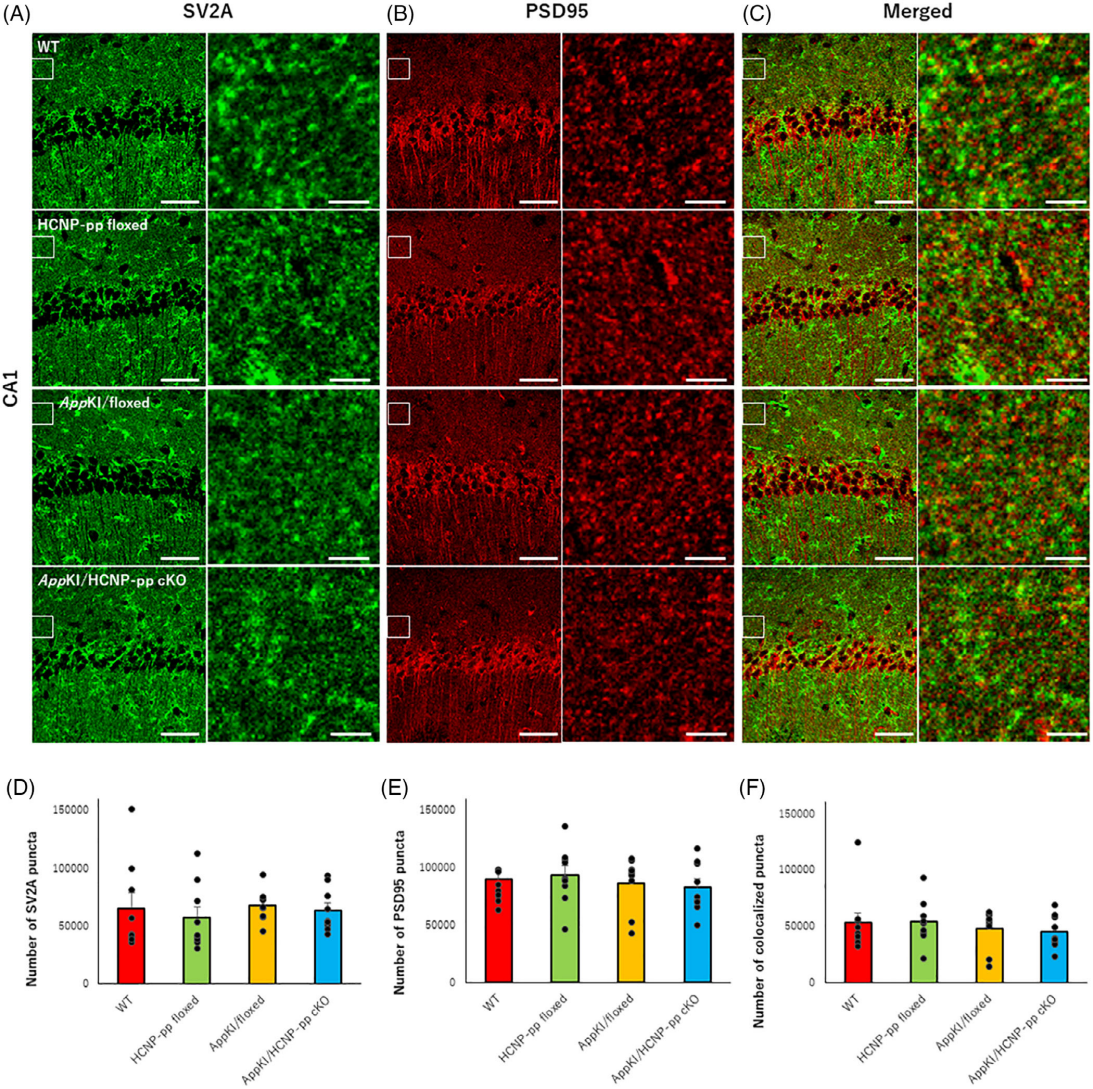
Evaluation of synaptic terminals. (A–C) Representative deconvoluted confocal *Z*‐stack images of the CA1 region stained with anti‐synaptic vesicle glycoprotein 2A (SV2A) and anti‐postsynaptic density 95 (PSD95) antibodies. (D–F) Quantification revealed no significant differences in the numbers of SV2A‐positive, PSD95‐positive, or SV2A/PSD95 double positive puncta (< 1 µm) in *App^NL‐G‐F^* KI/HCNP‐pp cKO mice compared with those in WT, HCNP‐pp‐floxed, and *App^NL‐G‐F^* KI/floxed mice. These data suggested that HCNP reduction does not significantly decrease synaptic terminal density in the CA1 region of *App^NL‐G‐F^* KI/floxed mice. Male mice aged 9–10 months were included in this study. *N* (mouse) = 3, *n* (slice) = 9. All data are presented as the mean ± S.E.M. Data were analyzed using a linear mixed‐effects model with genotype as a fixed effect and mouse identification document (ID) as a random intercept to account for multiple slices per mouse. *p < 0.05, **p < 0.01 (raw data and statistical details are provided in Table S9).

Synaptic loss and degeneration have been reported in *App^NL‐G‐F^* KI mice.^27^ Therefore, we next examined whether a reduction in synaptic terminals within the CA1 region contributes to the exacerbation of cognitive dysfunction observed in *App^NL‐G‐F^* KI/HCNP‐pp cKO mice. To quantify synaptic terminals, we analyzed deconvoluted confocal *Z*‐stack images obtained from the CA1 region following double immunostaining with anti‐SV2A and anti‐PSD95 antibodies. In the current experiment, no significant differences were observed in the numbers of SV2A‐positive, PSD95‐positive, or SV2A/PSD95 co‐localized puncta in *App^NL‐G‐F^* KI/HCNP‐pp cKO mice compared with those in *App^NL‐G‐F^* KI/floxed mice (Figure 7A–C, Table S9). These data suggested that HCNP reduction does not significantly decrease synaptic terminal density in the CA1 region of *App^NL‐G‐F^* KI/floxed mice.

### 3.7 No significant change in inflammatory pathology in *App^NL‐G‐F^* KI mice with reduced HCNP expression as assessed via astrocyte and microglial reactions

**FIGURE 8:**
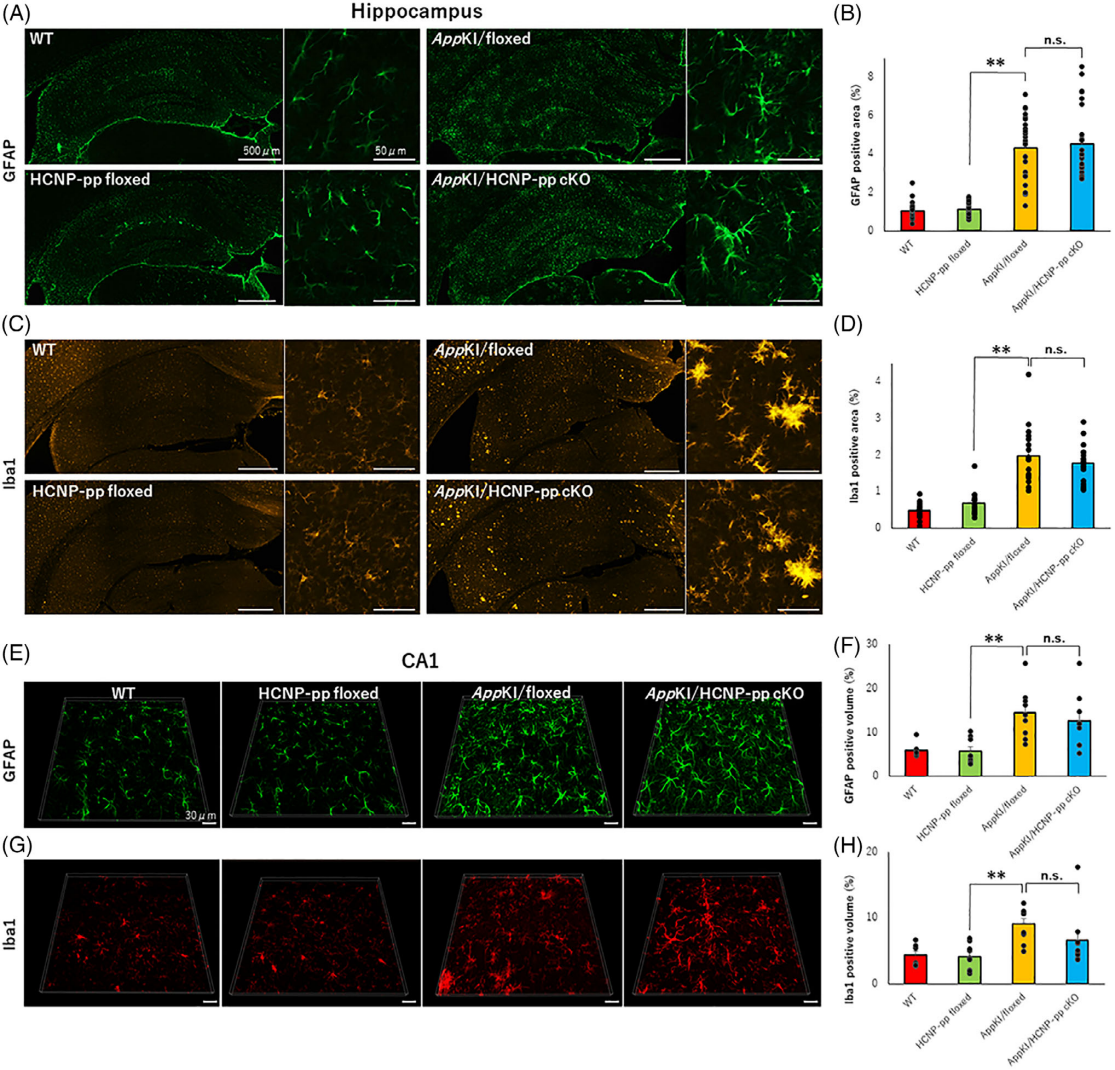
Evaluation of inflammation. (A, E) Glial fibrillary acidic protein (GFAP) immunostaining revealed larger astrocytes and increased processes in the *App^NL‐G‐F^* KI/floxed and *App^NL‐G‐F^* KI/HCNP‐pp cKO groups. Scale bar = 500 µm (left), 50 µm (right). (B, F) An increase in the GFAP‐positive area% in the hippocampus and volume% within the stratum oriens layer of the CA1 field was observed in the *App^NL‐G‐F^* KI/floxed and *App^NL‐G‐F^* KI/HCNP‐pp cKO groups; however, the difference was not significant. (C,G) Ionized calcium‐binding adaptor molecule 1 (Iba1) immunostaining revealed clustering and enlargement of microglia in the *App ^NL‐G‐F^* KI/floxed and *App^NL‐G‐F^* KI/HCNP‐pp cKO groups. Scale bar = 500 µm (left), 50 µm (right). (D, H)An increase was observed in the Iba1‐positive area% in the hippocampus and volume% within the stratum oriens layer of the CA1 field in the *App ^NL‐G‐F^* KI/floxed and *App^NL‐G‐F^* KI/ HCNP‐pp cKO groups; however, the difference was not significant. The data suggested that HCNP did not significantly affect inflammation. Male mice 9–10 months of age were used. *N* (mouse) = 4, *n* (slice) = 24. All data are presented as the mean ± S.E.M. Data were analyzed using a linear mixed‐effects model with genotype as a fixed effect and mouse identification document (ID) as a random intercept to account for multiple slices per mouse. *p < 0.05, **p < 0.01 (raw data and statistical details are provided in Table S10).

The exacerbation of inflammatory reactions in the hippocampus is involved in cognitive dysfunction in *App^NL‐G‐F^* KI mice.^20^ Therefore, we examined whether the inflammatory reaction is involved in the exacerbation of cognitive function in *App^NL‐G‐F^* KI/HCNP‐pp cKO mice. To address this, we assessed alterations in microglia and astrocytes by conducting immunohistochemical and volumetric analyses using Imaris software. We observed no significant exacerbation in the Iba1‐ and GFAP‐positive areas and volume of the hippocampus between *App^NL‐G‐F^* KI/HCNP‐pp cKO and *App^NL‐G‐F^* KI/floxed mice (Figure 8A–D, E–H; Table S10). These data suggested that the reduced HCNP level in the hippocampus of *App^NL‐G‐F^* KI mice did not significantly alter the inflammatory reaction, although qualitative changes in microglia and astrocytes cannot be excluded.

## 4 DISCUSSION

We found that reduced HCNP/ HCNP‐pp levels may exacerbate memory function in *App^NL‐G‐F^* KI mice; reduced HCNP/ HCNP‐pp levels may inhibit the fEPSP slope during LTP in *App^NL‐G‐F^* KI mice via cholinergic dysfunction from the MSN to the hippocampus, accompanied by reductions in ChAT, VAChT, and NR2A levels in the hippocampus and a decrease in ChAT levels in the cholinergic neurons of the MSN; and no significant acceleration was observed in Aβ accumulation, number of synaptic terminals, and inflammation driven by microglia or astrocytes in the hippocampus of *App^NL‐G‐F^* KI mice as a result of reduced HCNP/HCNP‐pp levels.

Our previous study did not find evidence of behavioral impairment in HCNP‐pp cKO mice in terms of memory cognition; however, decreased ACh concentration in the hippocampus and inhibited hippocampal glutamatergic activation via cholinergic dysfunction have been confirmed.^14^, ^15^, ^17^ Electrophysiological investigation of WT mice confirmed that the effect of the cholinergic agonist occurs only under insufficient glutamatergic activation when using Aβ oligomers.^18^, ^21^ Hippocampal glutamatergic inhibition was observed in *App^NL‐G‐F^* KI mice,^28^, ^29^ with notable memory impairment observed via NOR testing in 12‐month‐old mice, as reported previously (Figure S2A, B, Table3).^30^ *App^NL‐G‐F^* KI mice were expected to exhibit insufficient hippocampal glutamatergic activation, enabling reduced HCNP levels to behaviorally manifest as enhanced memory impairment in *App^NL‐G‐F^* KI mice. Our results suggested that HCNP may reach a workable level during hippocampal dysfunction. Modulation of neuronal networks, including cholinergic afferents from the MSN, may not function constantly during hippocampal activation. To investigate the function of each factor in the behavioral phenotype, especially via network modulation, utilizing an appropriate model to assess physiological functions, including effects of the environment on the functioning network, is necessary.

HCNP‐pp‐floxed mice displayed a tendency toward anxiety‐related behavior but no differences in cognitive functioning. We previously found no significant alteration in the anxiety‐related behavior of HCNP‐pp cKO mice compared with HCNP‐pp‐floxed mice. However, HCNP‐pp cKO mice were not compared with WT mice; thus, we reexamined the levels of HCNP‐pp expressed in HCNP‐pp‐floxed mice and found a slight decrease in the HCNP‐pp level in the hippocampus compared with that in WT mice; however, the extent of decline was minor compared with that observed in HCNP‐pp cKO mice (Figure S1A, B; Table S1). Reduced HCNP‐pp levels might induce anxiety‐related behavior; however, this effect was not demonstrated because HCNP‐pp cKO mice were not included as controls. The mechanism underlying HCNP‐pp reduction in HCNP‐pp‐floxed mice remains unknown; however, unintended loxP integration in the targeting vector used to generate HCNP‐pp‐floxed mice was excluded.^15^

Hippocampal glutamatergic activation is the primary neuronal activity related to learning and memory.^31^, ^32^ The pathways to the hippocampus that modulate glutamatergic activation include cholinergic and γ‐aminobutyric acid (GABA)ergic neurons from the MSN, serotonergic neurons from the raphe nucleus, noradrenergic neurons from the locus coeruleus, and dopaminergic neurons from the mammillary body.^33^, ^34^, ^35^, ^36^, ^37^ However, the conditions that induce neuronal activation in the modulating network and the extended interactions between them remain unclear. Regarding neuronal dysfunction in the AD brain, cholinergic dysfunction in the MSN may precede cholinergic cell death, and glutamatergic hyperexcitation may induce cognitive impairment by increasing the signal‐to‐noise ratio, resulting in glutamatergic neuronal cell death in the hippocampus.^38^, ^39^, ^40^ Cholinergic afferents may undergo selective degeneration under AD progression.^38^ Thus, AChE inhibitors may become less clinically effective owing to decreased ACh release.^41^, ^42^, ^43^ We demonstrated that reduced levels of HCNP may be directly involved in the electrophysiological and behavioral exacerbation in *App^NL‐G‐F^* KI mice. Reduced VAChT and ChAT levels in the hippocampus and decreased ChAT levels in the MSN were observed in *App^NL‐G‐F^* KI/HCNP‐pp cKO mice, similar to those observed in HCNP‐pp cKO mice.^16^

Decreased NR2A levels were observed in the *App^NL‐G‐F^* KI/HCNP‐pp cKO group; no significant alteration was observed in *App^NL‐G‐F^* KI or HCNP‐pp cKO mice.^16^ Activation of synaptic NMDA receptors promotes hippocampal function, with NMDA receptors presenting varying properties depending on their subunit composition: NR2A is mainly localized in the synaptic area, whereas NR2B localizes to the extrasynaptic area. Extracellular Aβ tends to co‐localize with NR2B subunits, advancing AD pathology via cAMP response element binding protein (CREB) dephosphorylation due to activation of extrasynaptic NR2B‐NMDA receptors.^44^, ^45^ Reduced NR2A levels may be involved in the exacerbation of hippocampal glutamatergic functioning in *App^NL‐G‐F^* KI/HCNP‐pp cKO mice, such as LTP; however, the mechanism underlying decreased NR2A levels remains unknown.

Atrophy of the basal forebrain cholinergic projections may precede entorhinal, and subsequently neocortical degeneration.^38^, ^46^ To physiologically preserve the cholinergic network as a modulator, the protection of cholinergic neurons in the basal forebrain is a conceivable disease‐modifying pharmacological approach. A potential mechanism for the degeneration of cholinergic neurons, Rab5‐mediated endosomal dysfunction through p38 activation, results in degeneration of cholinergic neurons via inhibition of nerve growth factor (NGF) signals.^47^ When inhibiting the binding of mature NGF to the tropomyosin‐related kinase A (TrkA) receptor, pro‐NGF signaling through p75 neurotrophin receptor (p75NTR) may lead to cholinergic degeneration in the basal forebrain.^48^ Reduced HCNP levels did not induce cholinergic neurodegeneration in *App^NL‐G‐F^* KI mice; however, ChAT levels decreased. HCNP may function complementarily with NGF to increase the ChAT levels, whereas HCNP administration into the ventricle electrophysiologically enhances cholinergic function with a decrease in TrkA and p75NTR levels.^22^, ^49^ Elucidation of the association between NGF and HCNP is required to inhibit the neurodegeneration of cholinergic neurons in the MSN.

Inhibition of cholinergic activation from the MSN to the hippocampus has also been reported in *App^NL‐G‐F^* KI mice.^9^ Conversely, cholinergic dysfunction may reduce the ability for Aβ clearance.^50^ In the present study, reduced HCNP levels exacerbated cognitive dysfunction without altering Aβ accumulation in *App^NL‐G‐F^* KI mice, indicating that the electrophysiological and behavioral phenotypes varied despite retaining a steady degree of APP pathological changes. This finding aligned with previous study results; several neuronal modulators have been observed to ameliorate motor function in patients with Parkinson's disease, presumably by modulating the dopaminergic network from the compact part of the substantia nigra to the corpus striatum without decreasing α‐synuclein protein accumulation.

Neuroinflammatory reactions have been observed in many neurodegenerative diseases.^51^ The inflammatory reaction was initially thought to be a secondary reaction to primary pathogenic changes. However, alteration of the immune system reportedly starts early by involving microglia and chemical mediators, and AD immunopathology is generated independently via Aβ aggregation and neurofibrillary tangles.^52^, ^53^ Specific disease‐associated microglia have been reported recently in AD under excitation with *APOE*, TREM2, CSF1, and *CST7*, followed by astrocyte activation.^53^, ^54^ Microglial and astrocyte activation have also been reported in combination with Aβ42 accumulation in the cortical region and hippocampus of *App^NL‐G‐F^* KI mice.^20^ In the present study, the reduced HCNP levels enhanced cognitive impairment in *App^NL‐G‐F^* KI mice without eliciting inflammation.

Lifestyle modification is considered a means of preventing dementia, resulting in resistance, resilience, and cognitive reserve.^55^ Cognitive training, motion exercise, and risk management of vascular events may help avoid cognitive function decline while improving brain resilience.^5^ The brains of SuperAgers, who are >80 years old but demonstrate memory performance at the level of 50‐ to 60‐year‐olds, show reduced AChE activity with several pathognomonic characteristics.^56^, ^57^, ^58^, ^59^ The present study indicated that cholinergic dysfunction may enhance cognitive impairment in *App^NL‐G‐F^* KI mice through HCNP reduction, without changes in APP pathology or Aβ pathology‐induced inflammation. Accordingly, individual variations in cognitive reserve or resilience may depend on the persisting ability to modulate neuronal activity and independent APP pathological changes.^60^, ^61^ To elucidate the potential of modulating systems, including cholinergic neurons from the MSN, serotonergic neurons from the raphe nucleus, noradrenergic neurons from the locus coeruleus, and dopaminergic neurons from the mammillary body, detailed pathological validation is needed.

This study had some limitations. First, we did not show whether overexpression or administration of HCNP or an AChE inhibitor, was able to rescue cognitive dysfunction attributed to HCNP reduction in *App^NL‐G‐F^* KI mice. In the future, we plan to conduct additional experiments to determine whether HCNP can rescue the behavioral phenotype in *App^NL‐G‐F^* KI/HCNP‐pp cKO mice, confirming the importance of cholinergic function through HCNP in the amelioration of cognitive dysfunction in the model. Second, we were unable to ascertain the cause for decreased NR2A levels in the hippocampus of *App^NL‐G‐F^* KI/HCNP‐pp cKO mice. The possibility of a type II error could not be completely excluded in the western blot, ELISA, and immunohistochemical analyses because of the small sample size; however, the estimates of effect sizes and confidence intervals suggest that large effects of genotype on the measured outcomes are unlikely to have been missed. Third, we could not confirm the degree of neurodegeneration of cholinergic neurons in the forebrain of aging individuals with cognitive reserves or resilience. To validate the importance of cholinergic functioning through HCNP in the mechanism by which cognitive resilience occurs, pathological analysis of an AD brain would be required. Finally, *App^NL‐G‐F^* KI mice showed typical amyloid aggregation along with inflammatory reactions, including microglia and astrocyte infiltration; however, no significant tau pathology was observed. Therefore, we could not explore the association between cholinergic reduction and tau pathology; additional experiments are required to determine the significance of cholinergic function in cognitive reserve or resilience.

## 5 CONCLUSION

This study demonstrated that reduced HCNP levels enhance the cognitive impairment in *App^NL‐G‐F^* KI mice without changes in APP pathology or Aβ pathology‐induced microglial and astrocytic responses. Nevertheless, the possibility of false negatives in the western blot, ELISA, and immunohistochemical analyses cannot be excluded because of the small sample size. Our study further established an Alzheimer's mouse model characterized by cholinergic dysfunction and amyloid pathology. This mouse model could be useful in further investigations of cholinergic dysfunction and amyloid pathogenesis in AD.

## CONFLICT OF INTEREST STATEMENT

The authors disclose receipt of the following financial support for the research, authorship, and/or publication of this article: Otsuka Pharmaceutical Co., Ltd. (Japan), Kyowa Kirin Co., Ltd. (Japan), Daiichi Sankyo Company (Japan), Eisai Company (Japan), and Dainippon Sumitomo Pharmaceutical Co., Ltd. (Japan) funded Noriyuki Matsukawa. Author disclosures are available in the Supporting Information.

## CONSENT STATEMENT

Consent was not applicable in this study because the experiments were performed using animals only.

## Supporting information




**Supporting Information**: alz71531‐sup‐0001‐tableS1.xlsx

**Supporting Information**: alz71531‐sup‐0002‐tableS2.xlsx

**Supporting Information**: alz71531‐sup‐0003‐tableS3.xlsx

**Supporting Information**: alz71531‐sup‐0004‐tableS4.xlsx

**Supporting Information**: alz71531‐sup‐0005‐tableS5.xlsx

**Supporting Information**: alz71531‐sup‐0006‐tableS6.xlsx

**Supporting Information**: alz71531‐sup‐0007‐tableS7.xlsx

**Supporting Information**: alz71531‐sup‐0008‐tableS8.xlsx

**Supporting Information**: alz71531‐sup‐0009‐tableS9.xlsx

**Supporting Information**: alz71531‐sup‐0010‐tableS10.xlsx

**Supporting Information**: alz71531‐sup‐0011‐figureS1.tif

**Supporting Information**: alz71531‐sup‐0012‐figureS2.tif

**Supporting Information**: alz71531‐sup‐0013‐figureS3.tif

**Supporting Information**: alz71531‐sup‐0014‐figureS4.tif

**Supporting Information**: alz71531‐sup‐0015‐SuppMat.pdf
