# Supplementary figures and images for: Reduction in hippocampal cholinergic neurostimulating peptide enhances memory impairment in AppNL‐G‐F KI mice
Source: Alzheimers Dement. 2026 Jun 3;22(6):e71531. doi: 10.1002/alz.71531 (PMC13240342; doi:10.1002/alz.71531)

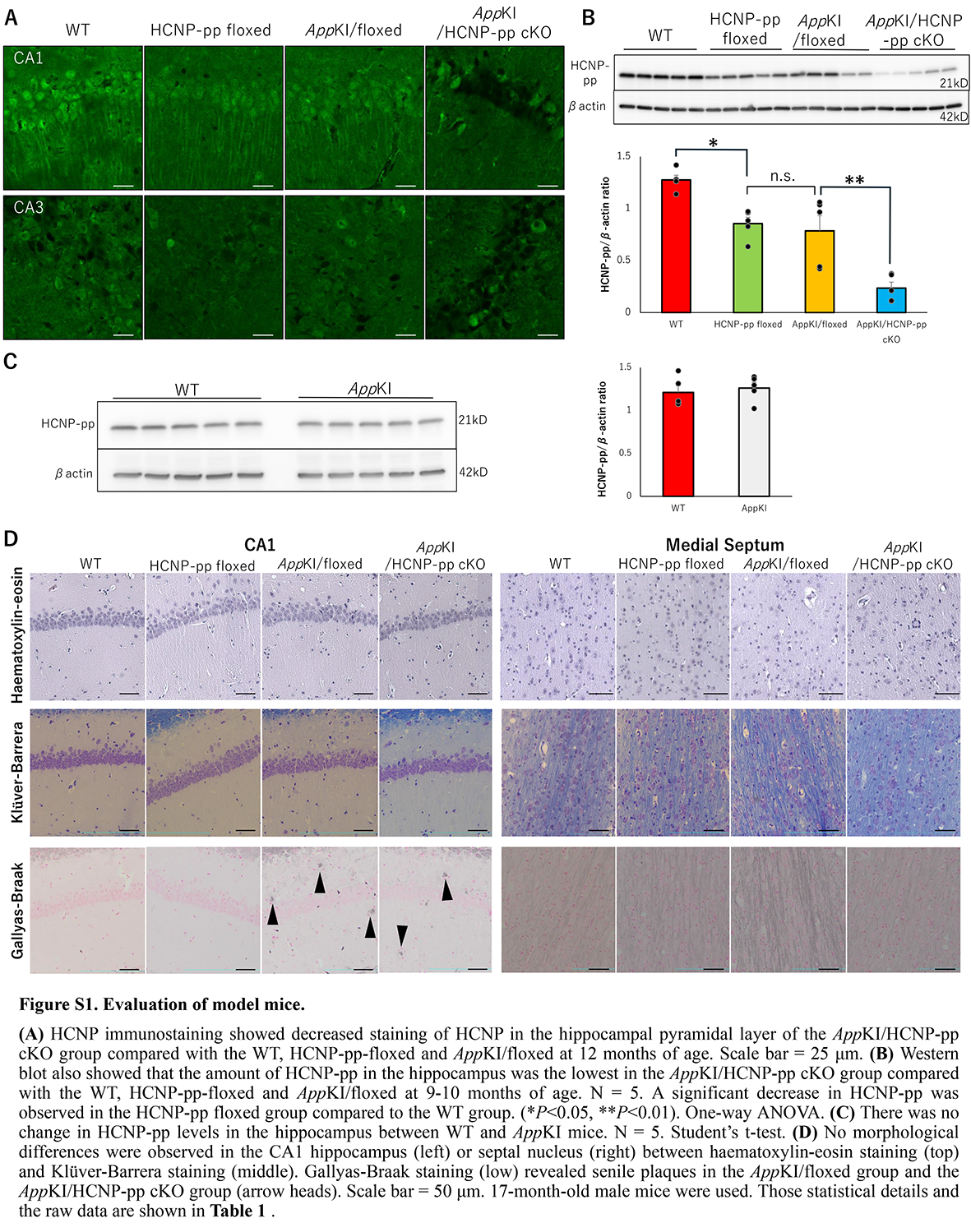

Supplement: Supplementary file 11 — Supporting Information: alz71531‐sup‐0011‐figureS1.tif [file ALZ-22-e71531-s003.tif]

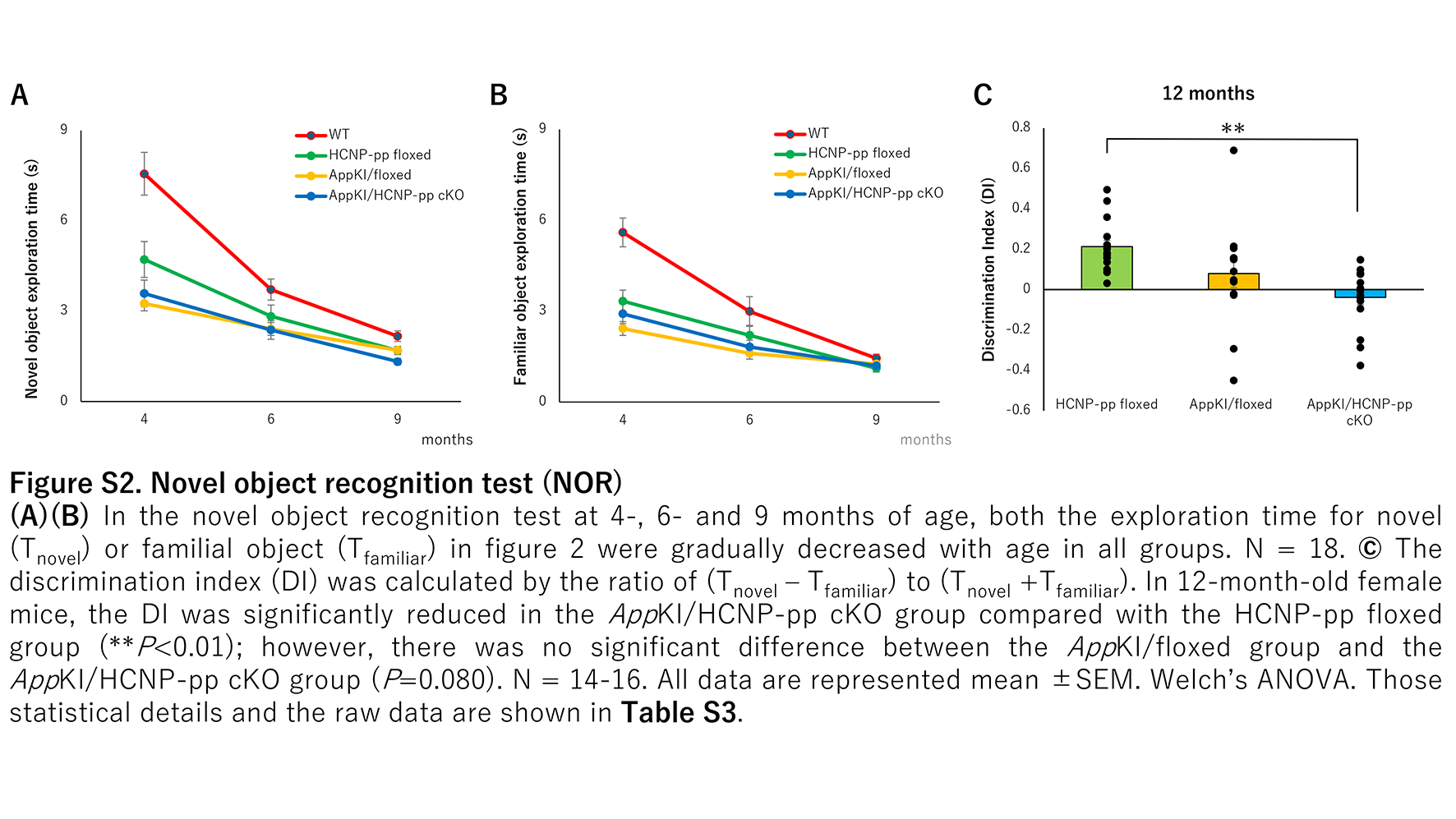

Supplement: Supplementary file 12 — Supporting Information: alz71531‐sup‐0012‐figureS2.tif [file ALZ-22-e71531-s015.tif]

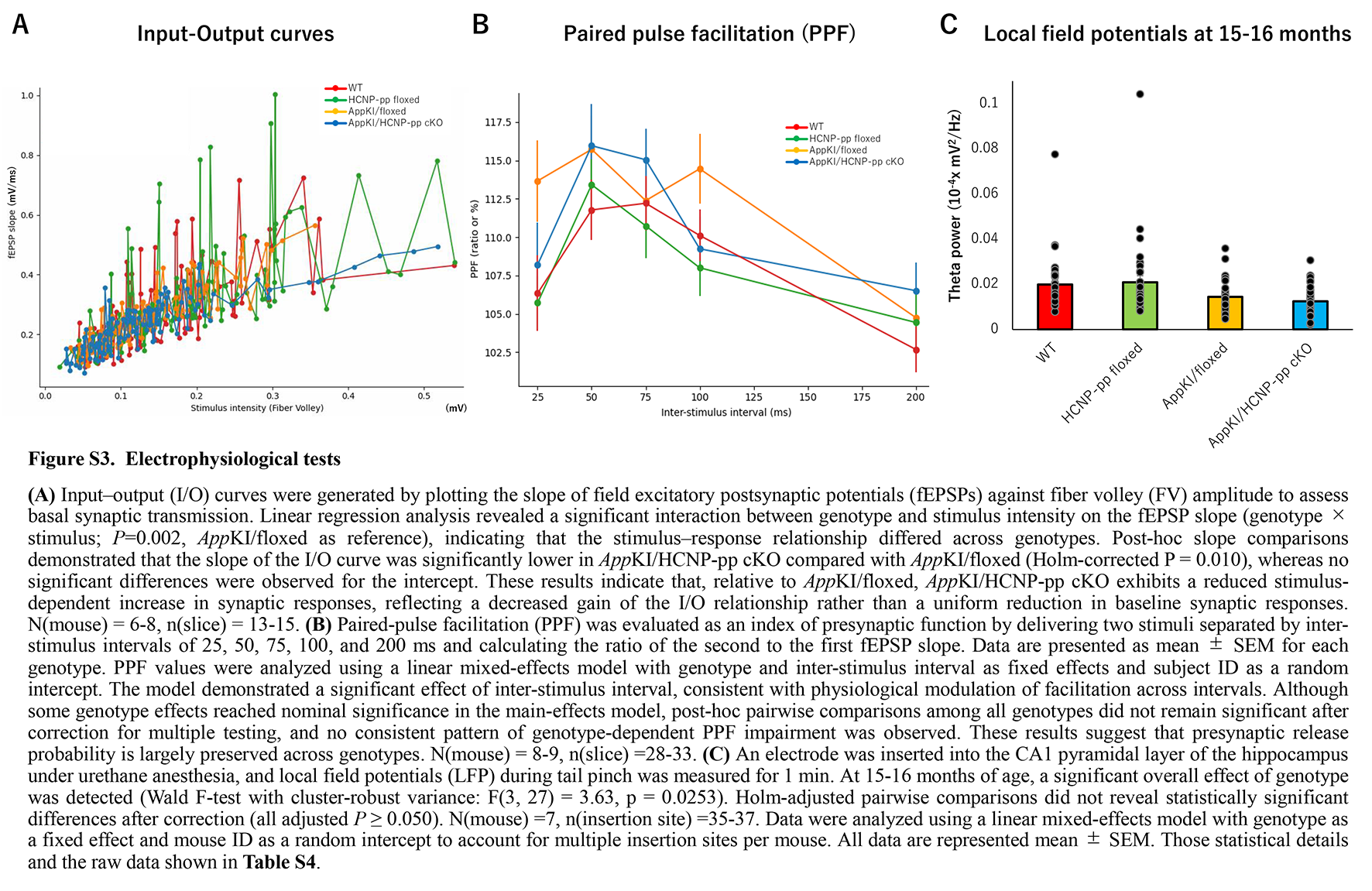

Supplement: Supplementary file 13 — Supporting Information: alz71531‐sup‐0013‐figureS3.tif [file ALZ-22-e71531-s008.tif]

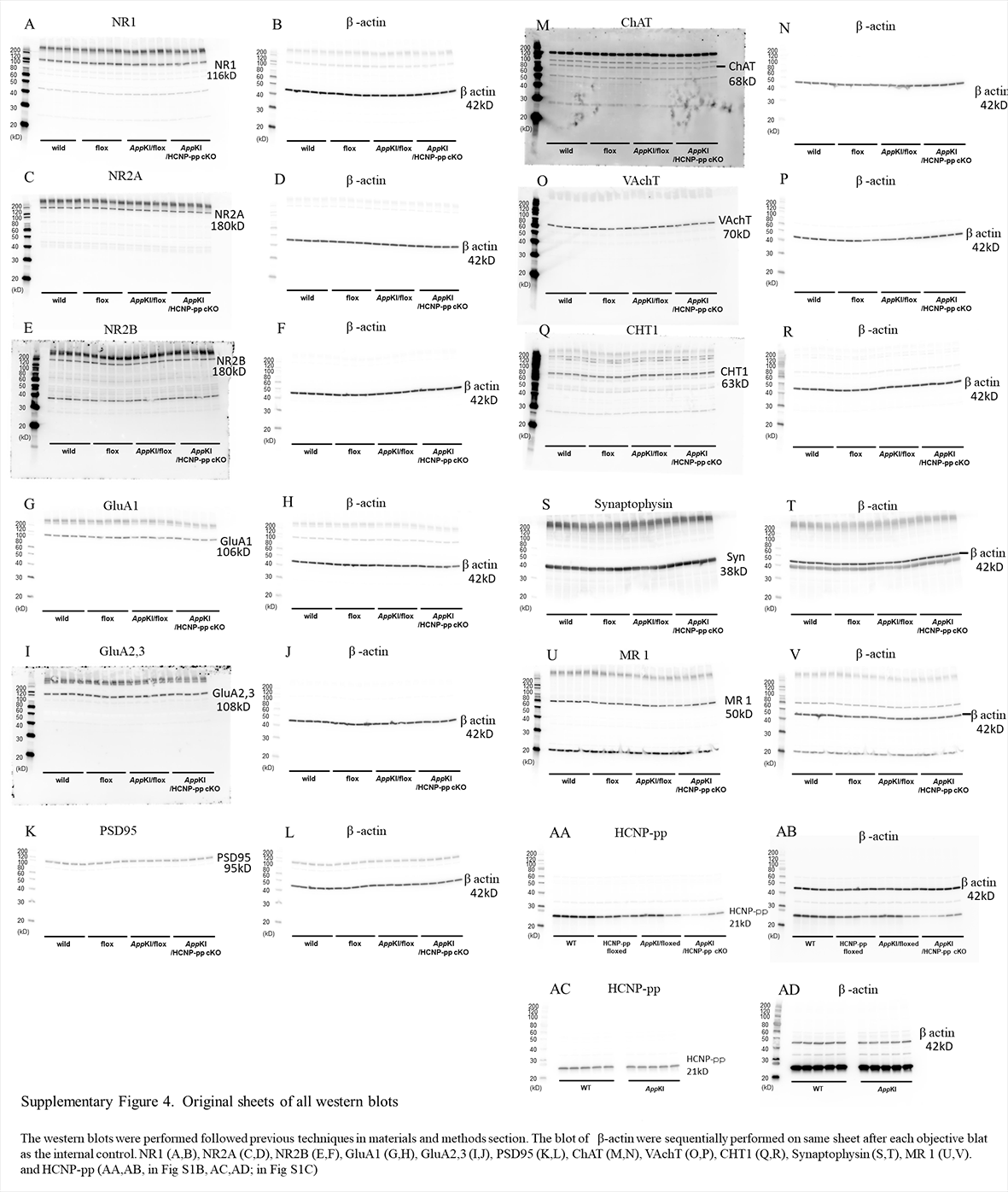

Supplement: Supplementary file 14 — Supporting Information: alz71531‐sup‐0014‐figureS4.tif [file ALZ-22-e71531-s013.tif]
